# Aspirin Enhances the Protection of Hsp90 from Heat-Stressed Injury in Cardiac Microvascular Endothelial Cells Through PI3K-Akt and PKM2 Pathways

**DOI:** 10.3390/cells9010243

**Published:** 2020-01-18

**Authors:** Xiaohui Zhang, Bixia Chen, Jiaxin Wu, Junzhou Sha, Bo Yang, Jie Zhu, Jiarui Sun, Jörg Hartung, Endong Bao

**Affiliations:** 1 College of Veterinary Medicine, Nanjing Agricultural University, Nanjing 210095, China; zhangxh@njau.edu.cn (X.Z.); 2019807112@njau.edu.cn (B.C.); 2018807111@njau.edu.cn (J.W.); 2018107002@njau.edu.cn (J.S.); 2019107002@njau.edu.cn (B.Y.); 2019107001@njau.edu.cn (J.Z.); 2016207004@njau.edu.cn (J.S.); 2 Institute for Animal Hygiene, Animal Welfare and Farm Animal Behaviour, University of Veterinary Medicine Hannover, 30559 Hannover, Germany; Joerg.Hartung@tiho-hannover.de

**Keywords:** heat stress, cardiac microvascular endothelial cells, Hsp90, signaling pathway, aspirin

## Abstract

Heat stress (HS) often causes sudden death of humans and animals due to heart failure, mainly resulting from the contraction of cardiac microvasculature followed by myocardial ischemia. Cardiac microvascular endothelial cells (CMVECs) play an important role in maintaining vasodilatation. Aspirin (ASA) is well known for its protective abilities of febrile animals. However, there is little knowledge about molecular resistance mechanisms of CMVECs and which role ASA may play in this context. Therefore, we used a heat stress model of rat cardiac microvascular endothelial cell cultures in vitro and investigated the cell injuries and molecular resistance mechanism of CMVECs caused by heat stress, and the effect of aspirin (ASA) on it. HS induced severe pathological damage of CMVECs and cellular oxidative stress and dysfunction of NO release. Hsp90 was proven to be indispensable for resisting HS-injury of CMVECs through PI3K-Akt and PKM2 signaling pathways. Meanwhile, PKM2 functioned in reducing Akt phosphorylation. ASA treatment of CMVECs induced a significant expression of Hsp90, which promoted both Akt and PKM2 signals, which are beneficial for relieving HS damage and maintaining the function of CMVECs. Akt activation also promoted HSF-1 that regulates the expression of Hsp70, which is known to assist Hsp90′s molecular chaperone function and when released to the extracellular liquid to protect myocardial cells from HS damage. To the best of our knowledge, this is the first study to show that HS damages CMVECs and the protection mechanism of Hsp90 on it, and that ASA provides a new potential strategy for regulating cardiac microcirculation preventing HS-induced heart failure.

## 1. Introduction

Excessive exposure to external heat (Heat stress, HS) can result in a severe life-threatening body heatstroke, a clinical condition characterized by a core temperature rising rapidly above 40 °C and central nervous system dysfunction [1]. Environmental heat stress is a serious cause of natural death and at least 7% of environment-related deaths worldwide [2]. A heat wave affected Europe in the summer of 2003 summer and led to 45,000 excess deaths, of which one-third was attributed to heat stress [3]. Global warming is predicted to increase the frequency and severity of heat stress, which is associated with a rise in mortality [3].

Accumulating evidence proves that high heat can stimulate cell death and tissue injury, especially in cardiac tissue [4,5]. Heat stress significantly increases the energy metabolism and oxygen demand of cardiac tissue. Myocardial cells are prone to increase ROS (reactive oxygen species) production in mitochondria due to insufficient oxygen supply, inducing heat stress-related heart failure [4]. Therefore, maintaining heart function requires a sustained and adequate supply of blood oxygen from cardiac microvasculature [4]. A former study has shown that transportation stress induces microvascular contractions in pig heart, resulting in myocardial ischemia and heart failure [6]. When an animal is in a stress state, the enhancement of cardiac contractile force, accompanied by a low nitric oxide level in blood, brings a serious burden to the blood supply of the cardiovascular system [7].

Cardiac microvascular endothelial cells (CMVECs) play an important role in ensuring the blood supply of myocardial tissue [8]. After exposure to diverse stressors, the endothelial cells serve not only as a barrier between the bloodstream and tissue parenchyma but also as transducing cells to modulate the parenchymal responses to stress [9]. Previous studies using cell lines and animal models have suggested that vascular endothelial cells are the first to be injured by several stimuli such as heat stress and that severe heat stress is prominently characterized by damaged endothelial cells [10,11,12]. However, considering the tissue-specific heterogeneity of microvascular endothelial cells [13], little is known about the pathogenesis and biological effects of heat stress on CMVECs, especially from the point of cellular morphology.

Some previous study showed the increase of ROS first appeared in the heat-stressed human umbilical vein endothelial cells (HUVEC), and then inositol 1,4,5-triphate receptor induced up-regulation of cytoplasmic Ca^2+^, and Caspase-9 and Caspase-3 activation, finally inducing apoptosis [14,15]. However, no study has addressed protective mechanisms in heat-stressed CMVECs. Cells can produce a group of highly conserved protective proteins, heat shock proteins (Hsps, such as Hsp90 and Hsp70), which contribute to repair damages quickly in case of emergency [16]. As a molecular chaperone, Hsps stabilize newly synthesized proteins, repair damaged proteins, and activate survival signaling proteins [17]. Hsps also play an active oxygen scavenging role in cytoplasm, mitochondria and endoplasmic reticulum [18]. Our previous animal studies demonstrated strong Hsp90 and Hsp70 positive signals could be detected in the endothelial cells of cardiac microvasculature in all heat-stressed hearts, and aspirin (ASA), an antipyretic analgesic classified to nonsteroidal anti-inflammatory drugs (NSAIDs), could induce higher signaling density of Hsp90 [6,19,20]. However, the involvement of Hsps in the protection of heat-stressed CMVECs, and the protective mechanism of ASA, still remain to be more specifically determined.

In this study, the rat cardiac microvascular endothelial cells (CMVEC) culture in vitro were subjected to heat stress, and then characteristics of pathological damage and the expression kinetics of Hsps in the heat-stressed CMVECs were studied. The role of ASA in preventing heat stress damage to cells in the possible interaction with Hsp90 was also examined.

## 2. Materials and Methods

### 2.1. Cell Culture, Treatments and Cell Viability Assay

Primary rat cardiac microvascular endothelial cells (CMVECs) were purchased from Hunan Fenghui Biotechnology Co., Ltd. (Changsha, Hunan, China). The cell isolation procedure was carried out as follows: Neonatal rats were euthanized by isoflurane and the hearts were removed under sterile conditions. The left ventricle of the heart was immersed in 75% ethanol for 20 s in order to devitalize epicardial and endocardial endothelial cells, and then digested in 0.5% (*w/v*) collagenase type I (Gibco, Grand Island, NY, USA) for 20 min and 0.125% (*w/v*) trypsin (Hyclone, Logan, UT, USA) for 10 min at 37 °C in a shaking bath. After the addition of Dulbecco’s modified Eagle’s medium (DMEM, Gibco), the samples were filtered with a 70-nm metal mesh filter followed by centrifugation for 10 min at 400× *g* and subsequently re-suspended in DMEM supplemented with 20% (*v/v*) fetal bovine serum, 100 U/mL penicillin and 100 µg/mL streptomycin. Then the supernatants were seeded in polystyrene flasks at 37 °C/5% CO_2_. Cells (passage 3) were used for experiments. The rat myocardial cell line, H9C2, was purchased from Shanghai cell bank of the Chinese Academy of Sciences. H9C2 cell line was used in the experiment for testifying the effect of extracellular Hsp70 on cardiac parenchyma cells. CMVECs and H9C2 cells were heat-stressed through fast transference to another CO_2_ incubator at 43 °C for the designed times.

### 2.2. SiRNA Transfection

Rat Hsp90 was knocked down using specific small interfering RNAs (SiRNAs). For this, SiRNAs specifically targeting Hsp90α mRNA (S1, S2 and S3) were obtained from Invitrogen (Shanghai, China) as follows:

S1: sense sequence 5’-GGAAGGAGCUGCACAUUAAdTdT-3’
  anti-sense sequence 5’-UUAAUGUGCAGCUCCUUCCdTdT-3’
S2: sense sequence 5’-GGUGUCAGUUACCAAAGAAdTdT-3’
  anti-sense sequence 5’-UUCUUUGGUAACUGACACCdTdT-3’
S3: sense sequence 5’-GACUUAAGUUUCAUCUUAAdTdT-3’
  anti-sense sequence 5’-UUAAGAUGAAACUUAAGUCdTdT-3’

CMVECs in the logarithmic growth phase were carefully selected and transfected with the indicated SiRNAs after they reached 70% confluency by using Lipofectamine RNAiMAX reagent (Vazyme, Nanjing, Jiangsu, China), according to the manufacturer’s method. The proficiency of SiRNA transfection was examined by performing Western blotting. After 2 days of incubation at 37 °C in a humidified atmosphere of 5% CO_2_, cells were exposed to heat stress or not for 5 h.

### 2.3. ASA Treatment

CMVECs were treated with: (1) 0, 0.01, 0.05, 0.1, 0.2, 0.5, and 1 mg/mL aspirin for 24 h, after which their viability was measured; (2) 1 mg/mL aspirin including equimolar sodium bicarbonate (or not) for 0, 0.5, 1, 2, and 4 h to evaluate Hsps expression and cellular state; and (3) 1 mg/mL aspirin for 2 h and then heat-stressed at 43 °C (or not) for 5 h to investigate intracellular protein expression.

### 2.4. GA/TR Treatment

CMVECs were treated firstly with triciribine (TR) of 0, 0.5, 1, 2.5, 5, 10, 25, 50, and 100 µM for 24 h, after which cell viability was measured. Next, geldanamycin (GA) of 0.1 µM, as in our previous study [21], and/or 50 µM TR (firstly dissolved in DMSO, then diluted with the culture medium mentioned above) were added into CMVECs for 14 h, and then cells of different treatments were exposed to heat stress of 5 h or not to evaluate the cellular state and protein expression.

### 2.5. Cell Viability

Cell viability was assessed by cell counting kit-8 (CCK-8, DOJINDO, Kumamoto, Japan) according to the manufacturer’s instructions.

### 2.6. Detection of Lactate Dehydrogenase (LDH), Lipid Peroxide (LPO), Malondialdehyde (MDA) and Nitric Oxide (NO)

LDH activities and NO levels in the cell culture supernatant were detected using the corresponding detection kits from Beyotime (Nanjing, Jiangsu, China) and Jiancheng (Nanjing, Jiangsu, China), respectively, according to the manufacturers’ protocols. The levels of intracellular LPO and MDA were analyzed with the corresponding assay kits from Angle Gene (Nanjing, Jiangsu, China), according to the methods of the manufacturer.

### 2.7. Observation of Cell Morphology

Cells for cytopathological observation were cultivated in climbing flake and treated as designed. Cells of the climbing flake were first observed with a light microscope and then fixed with 4% polyformaldehyde solution for Hematoxylin-Eosin (H. E.) staining, as described previously [22]. The cells for the observation in the transmission electron microscope (TEM, Hitachi, Tokyo, Japan) were cultured directly on dishes fixed with 2.5% glutaraldehyde after the experimental treatment and sent to the General Hospital of the Eastern War Zone of the Chinese People’s Liberation Army for ultrastructural pathological analysis.

### 2.8. Flow Cytometric Analysis of Cell Apoptosis

The detection was performed according to the manual of AnnexinV-FITC apoptosis detection kit (Vazyme, Nanjing, Jiangsu, China). Treated cells were collected with trypsin digestion, washed with ice-cold PBS (phosphate buffer solution) twice, and re-suspended in binding buffer containing 5 µL annexinV-FITC. After 10 min incubation in the dark at room temperature, cells were added into the reaction buffer containing 10 µL propidium iodide (PI). Then flow cytometric analysis was immediately performed to detect apoptosis.

### 2.9. Cell Protein Extraction and Western Blot

Tested cells were lysed using the cell lysis buffer (Cwbio, Beijing, China) containing 1% phenylmethylsulfonyl fluoride, and the protein content of the lysates was quantified using the BCA protein assay kit (Cwbio). Cell protein extraction was evaluated by performing Western blot analysis as previously described [21], with primary antibodies including Anti-Hsp90α (Proteintech, Rosemont, IL, USA), Anti-Hsp27, Hsp70, protein kinase B (Akt), phosphorylated Akt (p-Akt), M2 isoform of pyruvate kinase (PKM2), Heat shock factor -1 (HSF-1), and Cytochrome C (Cyt-C, ZENBIO, Chengdu, Sichuan, China). HRP-conjugated second antibodies (Abbkine, Wuhan, Hubei, China) were used. Finally, reactive bands were quantified using the Quantity One software (ver. 4.6.2; Bio-Rad Laboratories, Hercules, CA, USA). β-actin was used as the loading control.

### 2.10. Immunofluorescence Analysis

CMVECs for immunofluorescence staining were fixed with 4% polyformaldehyde. The cells were washed with PBS twice, subsequently permeabilized in 0.5% Triton X-100 following blocking with 5% BSA for 30 min. Cells were incubated with primary antibodies for Akt plus Hsp90α, PKM2 plus Hsp90α, and Hsp70 plus Hsp90α at 4 °C overnight, and then incubated with rhodamine (plus fluorescein isothiocyanate)-conjugated secondary antibodies at 37 °C for 1 h. Cells were washed with PBS again before being mounted onto the slide with a mounting solution containing 0.2 mg/mL DAPI. Photographs of cells were taken under a fluorescence microscope.

### 2.11. Enzyme Linked Immunosorbent Assay (ELISA) Analysis for Hsp70 in Culture Medium

Hsp70 levels in culture medium were detected using special EILSA kit (Angle Gene) according to the manufacturer’s instructions.

### 2.12. Statistical Analysis

All data were analyzed for statistical significance using SPSS16.0 software (IBM, Chicago, IL, USA) with the analysis of One-way ANOVA. Data were expressed as means ± SD from at least three independent experiments performed in duplicate. *p* < 0.05 was considered statistically significant.

## 3. Results

### 3.1. Heat Stress Damaged CMVECs

**Figure 1:**
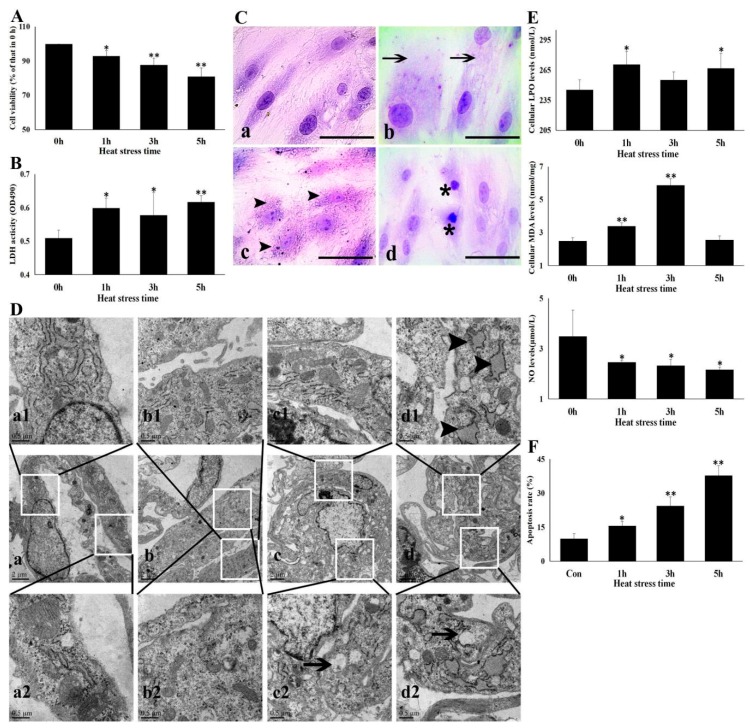
Heat stress damaged CMVECs. The CMVECs were exposed to heat stress for different amounts of time to observe cell injury. Data represent the means ± SD for three independent experiments. (**A**) CCK-8 test was used to detect cell viability. (**B**) LDH levels in the cell supernatant were analyzed to observe the cytomembrane injury. (**C**) Cytopathological observations were conducted under the light microscope. a–d show the morphology of cells exposed to heat stress of 0 h, 1 h, 3 h, and 5 h, respectively, after hematoxylin eosin (H. E.) staining. Bar = 20 µm. Arrows indicate swelling cells, arrowhead points to degeneration and loss of cytoplasm, and asterisk mark necrosis; (**D**) Ultrastructural damage of CMVECs was observed using TEM. a–d show the overall appearance of cells exposed to heat stress of 0 h, 1 h, 3 h, and 5 h, respectively; a1–d1 show details of the endoplasmic reticulum; and a2–d2 show the changes of the mitochondrion. Bar = 0.5 µm. (**E**) Specific kits were used to detect cellular oxidative stress levels and NO release in the supernatant. (**F**) Flow cytometry was used to detect the cellular apoptosis rate at different heat stress times. The differences of the data of cells with different heat stress times vs. that of the non-stressed cells are indicated by * *p* < 0.05 and ** *p* < 0.01.

To investigate the effect of HS on CMVECs, CCK-8 was firstly used to investigate the effect of HS on CMVEC viability (Figure 1A). HS at 43 °C significantly decreased CMVEC’s viability. Cell viability dropped after 1 h of HS by about 7.1%, after 5 h the drop in viability was 19%. At the same time, a higher LDH activity was found in the supernatant of the heat-stressed cell cultures, especially at 5 h (Figure 1B). An observation of the light microscope showed extreme swelling of CMVECs at 1 h of HS. After 3 h of HS, granular degeneration and vacuolization in cytoplasm were observed, accompanied by a partial loss of cytoplasm. Cell necrosis (karyopyknosis) could be found at 5 h of HS (Figure 1C). TEM observation in Figure 1D showed that at 1 h of HS, cell swelled, accompanied by a few enlarged endoplasmic reticulum and some swelled mitochondria whose cristae fell off and tangled. After heat stress for 3 h, the cell volume recovered; the endoplasmic reticulum cavity increased; and many mitochondrial cristae fell off, tangled, and even disappeared. At 5 h, the electron density of the nucleus chromatin increased, most of the cellular endoplasmic reticulum was extremely swollen and the lumen was enlarged, and many mitochondria changed into vacuoles due to the abscission of cristae.

As shown in Figure 1E, heat stress induced a significant increase of intracellular LPO and MDA levels, indicating that oxidative stress was stimulated by HS. We also found that NO released from heat-stressed CMVECs to supernatant was significantly decreased from 1 h of HS, compared with control cells (0 h of HS). Subsequently, heat-stressed apoptosis of CMVECs also increased sharply in a time-dependent manner (Figure 1F). These results indicate that HS severely damaged homeostasis and disturbed the function of CMVECs.

### 3.2. Hsp90 Expression were Up-Regulated in Heat Stressed CMVECs

**Figure 2:**
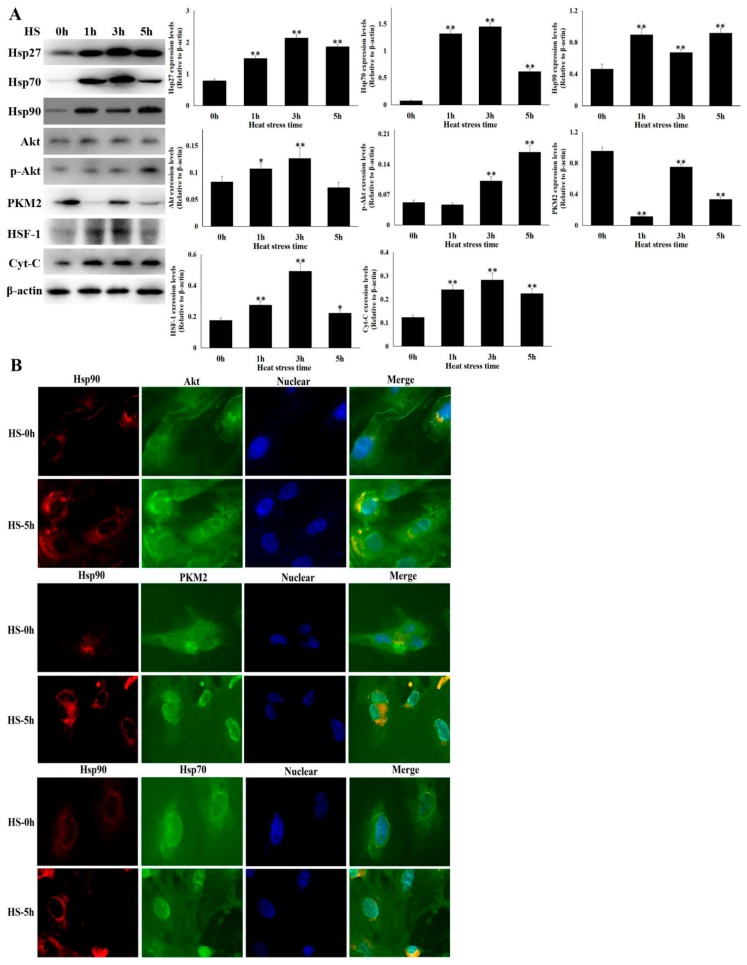
Hsp90 was up-regulated and affected its client proteins in heat-stressed CMVECs. Western blot analysis and immunocytochemistry were performed with the indicated antibodies. Data represent the means ± SD for three independent experiments. (**A**) CMVECs were treated with or without heat stress of the designed times. Total proteins were used for semi-quantitative detection of the corresponding proteins. The relative abundance of the tested proteins was normalized to that of β-actin. (**B**) CMVECs were treated with or without heat stress of the designed times. Immunofluorescence was performed using anti-Hsp90α, anti-Akt, anti-PKM2, and anti-Hsp70 antibodies. Representative images are presented (Hsp90: red fluorescence, Akt/PKM2/Hsp70: green fluorescence, nucleus: blue fluorescence, the merged signals of Hsp90 with Akt/PKM2/Hsp70 in the cytoplasm: yellow fluorescence). Bar = 20 µm. The differences of the data of cells with different heat stress times vs. that of the non-stressed cells are indicated by * *p* < 0.05 and ** *p* < 0.01.

To investigate the potential molecular protective mechanism, we firstly detected the expression of three critical heat shock proteins (Figure 2A). Hsp27, Hsp70, and Hsp90 were expressed in the non-treated CMVECs, and all were induced significantly by exposure to heat stress. Then, the key proteins of the survival signaling pathway related to Hsp90 were especially concerned (Figure 2A). We found that regarding the client proteins of Hsp90, Akt level was stimulated from 1 h of heat stress and came back to the control level at 5 h, while p-Akt increased from 3 h of heat stress, which was essential for cells to resist heat stress. Heat shock factor-1 (HSF-1), as a downstream molecule of p-Akt and regulator of Hsp70 expression [23], was also induced during the process of heat stress. However, another newly defined client protein of Hsp90, PKM2 [24], was decreased significantly. The molecule related to cellular apoptosis, Cyt-C, was induced in the whole process of heat stress, which was in agreement with the results of apoptosis rate during heat stress. The complex of Hsp90 with Akt and PKM2 caused by physical interaction directly mediates the stability and activation of Akt and PKM2, which are indispensable for regulating their downstream survival proteins, such as HSF-1 and Bcl-2, which is critical for their biological functions in regulating glycolysis, mitochondria respiration and apoptosis [23,24,25,26,27]. In addition, Hsp70 can assist the process through integrating with these complexes [23]. Therefore, cellular immunofluorescence was used to further observe the co-localization of the interaction and its sub-cellular distribution of Hsp90 plus Akt, Hsp90 plus PKM2, and Hsp90 plus Hsp70 in CMVECs. As shown in Figure 2B, after heat stress, the co-localization signals of Hsp90 with Akt, PKM2 and Hsp70 were all strengthened, and these signals of the merger were centralized around the nuclear. The results illustrate that Hsp90 is probably involved in resisting heat-stress injury of CMVECs through affecting its client proteins, Akt and PKM2, and then their downstream molecules.

### 3.3. Knockdown of Hsp90 Aggravated the Cellular Damage Induced by Heat Stress

**Figure 3:**
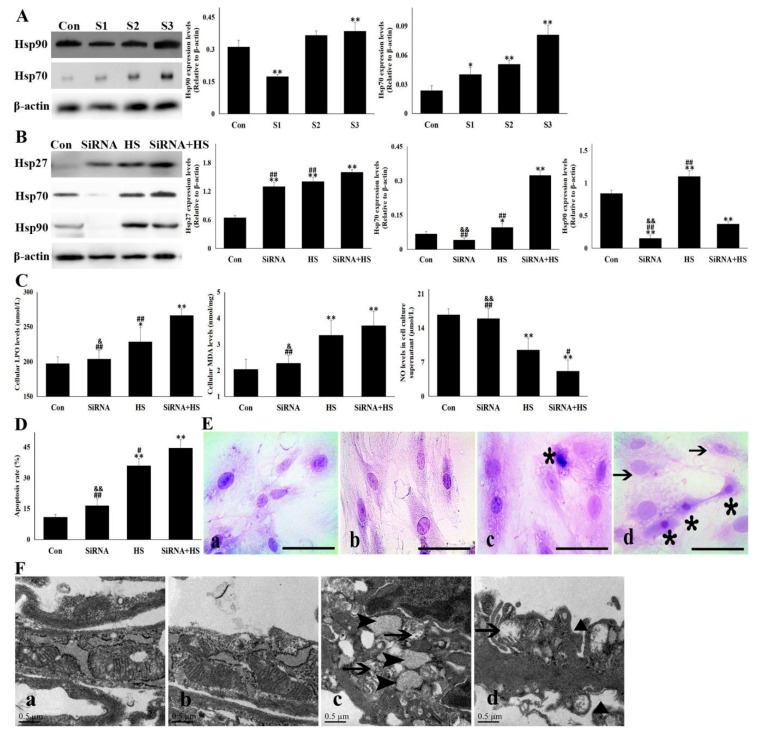
Knockdown of Hsp90 aggravated the cellular damage induced by heat stress. Data represent the means ± SD for three independent experiments. (**A**) CMVECs were transfected with a three SiRNA sequence (S1–S3) to inhibit Hsp90 expression and were harvested for detecting Hsp90 and Hsp70 levels with Western blot. The relative abundance of the tested proteins was normalized to that of β-actin. (**B**–**F**) CMVEC cells with or without Hsp90-SiRNA transfection were treated with heat stress for 5 h and harvested for corresponding detection: (**B**) Hsp27, Hsp70 and Hsp90 levels with Western blot analysis. The relative abundance of the tested proteins was normalized to that of β-actin. (**C**) The specific kits were used to detect cellular oxidative stress levels and NO release in the supernatant. (**D**) Flow cytometry was used to detect the cellular apoptosis rate. (**E**) H.E. staining was conducted and observed under the light microscope. a–d shows the representative pictures from Con, SiRNA, HS, and SiRNA + HS, respectively. Bar = 20 µm. Arrows point to the loss of cytoplasm, and asterisk mark necrosis. (**F**) Ultrastructural damage of CMVECs was observed using TEM. a–d shows the representative pictures from Con, SiRNA, HS, and SiRNA + HS, respectively. bar = 0.5 µm. Arrows indicate excessively swollen mitochondria, asterisk point to ballooning endoplasmic reticulum, and a black triangle marks the depressed or indented cytomembranes. Con, cells without SiRNA transfection and heat stress; SiRNA, cells transfected with SiRNA; HS, heat-stressed cells without SiRNA transfection; SiRNA + HS, heat-stressed cells following SiRNA transfection. The differences of the data of cells with different treatments vs. that of the Con are indicated by * *p* < 0.05 and ** *p* < 0.01. The differences of the data of cells treated by SiRNA + HS vs. that in SiRNA or HS are indicated by # *p* < 0.05 and ## *p* < 0.01. The difference of the data of cells treated by SiRNA vs. that in HS is indicated by & *p* < 0.05 and && *p* < 0.01.

To further confirm the involvement of Hsp90 in heat stress-induced injury, Hsp90 knockdown with SiRNA interference was conducted. As shown in Figure 3A, of the three tested SiRNA target sequences, S1 sequence was most effective in decreasing Hsp90 protein levels, accompanied by the increased expression of Hsp70. Therefore, S1 sequence was selected for subsequent study. After the exposure of SiRNA-transfected cells to heat stress for 5 h, the Hsp90 level was significantly lower than that in HS alone, which was also accompanied by s high expression of Hsp27 and Hsp70 (Figure 3B). Compared with non-treated cells, SiRNA-transfected cells were more susceptible to heat stress and are characterized by a larger increase in oxidative stress markers such as LPO and MDA, accompanied by lower viability (data not shown) and higher apoptosis rate (Figure 3C,D) during heat-stress. The loss of NO secretion into the supernatant was also aggravated in siRNA-transfected cells after heat stress, compared with that in HS (Figure 3C). An observation of cellular morphology also showed that the knockdown of Hsp90 induced more serious cellular damage characterized by more cellular necrosis and cytoplasmic loss (Figure 3E), and ballooning endoplasmic reticulum and mitochondria, including s depressed or indented cytomembrane (Figure 3F). These results confirm that Hsp90 plays an indispensable role in resisting heat-stress injury of CMVECs.

### 3.4. Akt and PKM2 were Regulated Differentially by Hsp90

**Figure 4:**
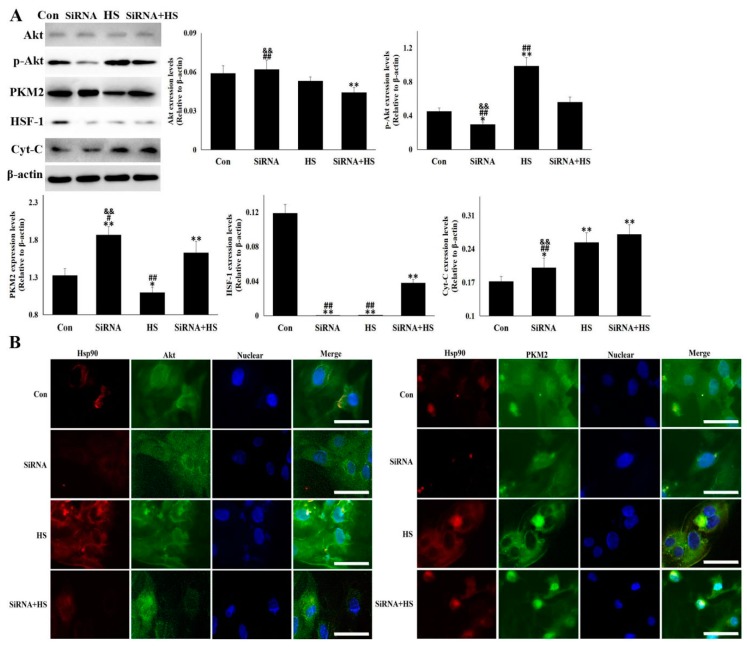
Akt and PKM2 were regulated differently by Hsp90. Western blot analysis and immunocytochemistry were performed with the indicated antibodies. Data represent the means ± SD for three independent experiments. (**A**) CMVECs were treated with or without SiRNA followed by heat stress of 5 h or not. Total proteins were used for semi-quantitative detection of the corresponding protein. The relative abundance of the tested proteins was normalized to that of β-actin. (**B**) CMVECs were treated with or without SiRNA followed by heat stress for 5 h or not. Immunofluorescence was performed using anti-Hsp90, anti-Akt and anti-PKM2 antibodies. Representative images were presented from Con, SiRNA, HS, and SiRNA + HS (Hsp90: red fluorescence, Akt/PKM2: green fluorescence, nucleus: blue fluorescence, the merged signals of Hsp90 with Akt/PKM2 in the cytoplasm: yellow fluorescence). Bar = 20 µm. Con, cells without SiRNA transfection and heat stress; SiRNA, cells transfected with SiRNA; HS, heat-stressed cells without SiRNA transfection; SiRNA + HS, heat-stressed cells following SiRNA transfection. The differences of the data of cells with different treatment vs. that of the Con are indicated by * *p* < 0.05 and ** *p* < 0.01. The differences of the data of cells treated by SiRNA+HS vs. that in SiRNA or HS are indicated by **#** *p* < 0.05 and **##** *p* < 0.01. The difference of the data of cells treated by SiRNA vs. that in HS is indicated by & *p* < 0.05 and && *p* < 0.01.

Based on evidence for the crucial role of Hsp90-mediated cellular protection in the process of heat stress, the client proteins of Hsp90 in the cell survival pathway were analyzed in SiRNA-transfected cells. As displayed in Figure 4A, Hsp90 knockdown with SiRNA interference decreased the Akt levels of the heat-stressed cells and also caused the obstacles of p-Akt generation, regardless of heat stress. At this time, PKM2 was up-regulated significantly by exposure to heat or not. Hsp90 knockdown or heat stress alone exacerbated the consumption of HSF-1, while both together partially recovered the HSF-1 level, which is responsible for Hsp70 rebound in the SiRNA + HS group. Hsp90 knockdown or heat stress alone increased the Cyt-C level to varying degrees, and both together further exacerbated the rise a little. Cellular immunofluorescence in Figure 4B shows that a loss of Hsp90 protein by SiRNA-mediated silencing obviously reduced the co-localization of Hsp90 with Akt and PKM2 (including Hsp70, data not shown) in the cells exposed to heat stress or not, but Hsp90 knockdown strengthened the residual merge signals of Hsp90 plus PKM2 and Hsp90 plus Hsp70 (like that of Hsp90 plus PKM2, data not shown) into the nuclear under heat stress. These data indicate that Akt activation was more sensitive to the cellular Hsp90 level, and that PKM2, as an alternative pathway of Akt, played a role in urgent cell protection under the condition of low Hsp90 expression.

### 3.5. PKM2 is a Alternative Pathway Under the Deficiency of Hsp90 Function and Akt Phosphorylation

**Figure 5:**
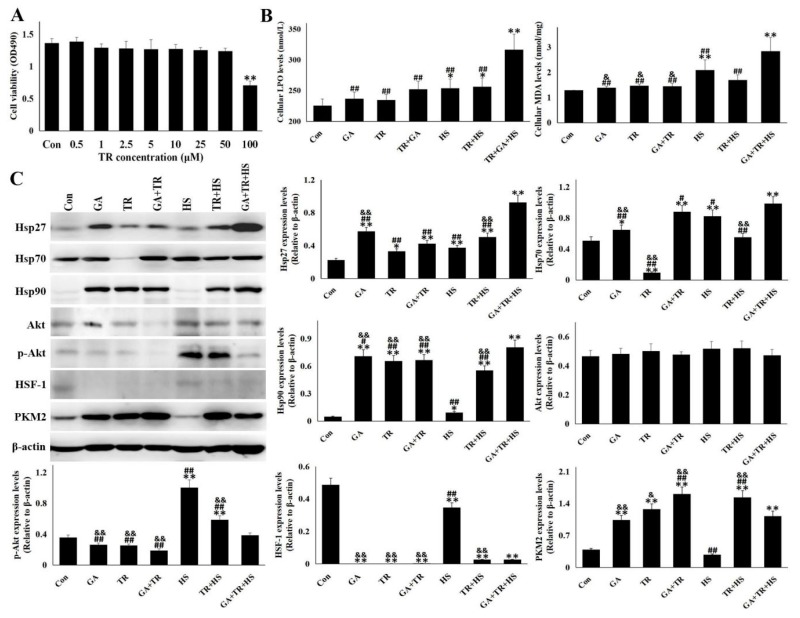
PKM2 was an alternative pathway under the deficiency of Hsp90 function and Akt phosphorylation. Data represent the means ± SD for three independent experiments. (**A**) After treatment with triciribine of different concentrations for 24 h, CCK-8 tests were used to detect cell viability. (**B**) After treatment with triciribine of 50 µm for 14 h, cells were exposed to heat stress for 5 h or not for detecting LPO and MDA levels. (**C**) After treatment with triciribine of 50 µm and/or 0.1 µM GA for 14 h, cells were exposed to heat stress for 5 h or not. Western blot analysis was used to detect the levels of different proteins with the indicated antibodies. Representative bands are presented in the left panel. Reactive bands were quantified using the Quantity One software, and the relative abundance of the tested proteins was normalized to that of β-actin (right panel). The differences of the data of cells with different treatment vs. that of the Con are indicated by * *p* < 0.05 and ** *p* < 0.01. The differences of the data of cells treated by TR + GA + HS vs. that in other groups are indicated by **#** *p* < 0.05 and **##** *p* < 0.01. The differences of the data of cells in HS vs. that in other groups are indicated by & *p* < 0.05 and && *p* < 0.01.

For further revealing the PKM2 pattern under the deficiency of Hsp90 function and Akt phosphorylation, 0.1 µM GA was used to block the function of Hsp90, which was verified by the up-regulation of Hsp70 [21]. Meanwhile, triciribine was used to inhibit Akt activation. Data in Figure 5A showed that TR of 100 µM could restrict the viability of tested cells, and the concentration of 50 µM was used in the inhibition study of Akt activation, which was confirmed through a subsequent Western blot. GA and TR treatment alone or together did not significantly influence the cellular oxidative state, but subsequent heat stress stimulated the cellular LPO and MDA levels. TR treatment followed by heat stress also induced oxidative stress characterized by LPO increase. Co-treatment with GA and TR could stimulate a greater intensity of LPO and MDA production than heat stress and TR plus HS (Figure 4B). Western blot in Figure 5C shows that GA and/or TR induce up-regulation of Hsp90, and subsequent heat stress further enhances the induction, especially for co-treatment with GA and TR. Under the condition of inhibition of Hsp90 function and/or Akt activation, both Hsp27 and Hsp70 expression were up-regulated to different degrees, especially for heat-stressed cells administrated with GA plus TR. Meanwhile, HSF-1 was down-regulated, which was reversed partially after exposure to heat stress. Interestingly, once Hsp90 function and/or Akt activation was blocked, a sharp increase of PKM2 levels was observed, with the decreased p-Akt level, regardless of heat-stress. These data demonstrate that deficiency of the Hsp90 function and/or an obstacle of Akt phosphorylation could induce the “Hsp90 level-miscalculated” and/or “p-Akt-compensative” high expression of PKM2, implying that Hsp90 only regulates the function of PKM2 and has little effect on its intracellular quantity, and that compensative expression of Hsp27 and Hsp70 caused from heat-induced HSF-1 would remedy the deficiency of Hsp90 function.

### 3.6. Hsp90 Induction by Aspirin Relieved the Heat-Stressed Damage

**Figure 6:**
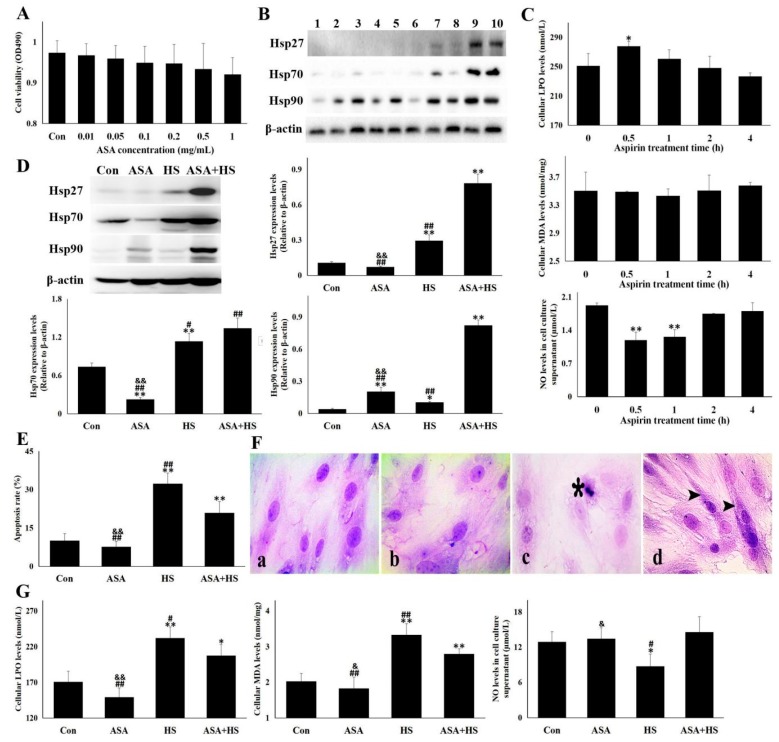
Hsp90 induction by aspirin relieved the heat-stressed damage. Data represent the means ± SD for three independent experiments. (**A**) After treatment with aspirin of different concentrations for 24 h, CCK-8 was used to detect cell viability. (**B**) CMVECs were administrated with aspirin of 1 mg/mL or aspirin plus sodium bicarbonate (ASA: 1 mg/mL; the mole ratio of ASA and NaHCO_3_ = 1:1) for different amounts of time. Hsp27, Hsp70 and Hsp90 levels were analyzed with Western blot analysis. The relative abundance of the tested proteins was normalized to that of β-actin. 1: ASA for 0 h; 2. ASA for 0.5 h; 3. ASA for 1 h; 4. ASA for 2 h; 5. ASA for 4 h; 6. ASA and NaHCO_3_ (ASA-Na) for 0 h; 7. ASA-Na for 0.5 h; 8. ASA-Na for 1 h; 9. ASA-Na for 2 h; and 10. ASA-Na for 4 h; (**C**) CMVECs were administrated with aspirin of 1 mg/mL for different time. The specific kits were used to detect cellular LPO and MDA levels and NO release into the supernatant. (**D–G**) CMVECs with or without aspirin treatment for 2 h were treated with heat stress for 5 h and harvested for corresponding detection. (**D**) Hsp27, Hsp70 and Hsp90 levels were analyzed with Western blot analysis. The relative abundance of the tested proteins was normalized to that of β-actin. (**E**) Flow cytometry was used to detect the cellular apoptosis rate. (**F**) H. E. staining was conducted and observed under the light microscope. a–d shows the representative pictures from Con, ASA, HS, and ASA+HS, respectively. Bar = 20 µm. Asterisk point to necrosis, and arrowhead mark degeneration. (**G**) The specific kits were used to detect cellular LPO and MDA levels and NO release into the supernatant. Con, cells without aspirin treatment and heat stress; ASA, cells treated with aspirin; HS, heat-stressed cells without aspirin; ASA + HS, heat-stressed cells following aspirin treatment. The differences of the data of cells with different treatment vs. that of the Con are indicated by * *p* < 0.05 and ** *p* < 0.01. The differences of the data of cells treated by ASA + HS vs. that in ASA or HS are indicated by **#** *p* < 0.05 and **##** *p* < 0.01. The difference of the data of cells treated by ASA vs. that in HS are indicated by & *p* < 0.05 and && *p* < 0.01.

Aspirin (ASA) is an effective anti-fever drug, however, little is known about its role and mechanisms in the resistance of CMVECs to heat stress. In this study, we tested whether ASA could serve as an agent to resist heat stress through the induction of Hsp90. Results in Figure 6A,B show that aspirin alone could only stimulate Hsp90 expression rather than Hsp27 and Hsp70, and did not significantly influence cell activity. In order to reveal whether the induction was from the acidity of ASA, the effect of aspirin plus sodium bicarbonate (ASA-Na) was also analyzed. ASA-Na showed a more broad-spectrum induction on not only Hsp90, but also Hsp27 and Hsp70. For more attentive investigation of the mechanism, ASA alone was used in the next study. Treatment of aspirin of 1 mg/mL for 2 h did not alter cellular oxidative stress levels and kept NO secretion normal (Figure 6C). After aspirin treatment as above, CMVECs were exposed to heat stress. Hsp90 in ASA-treated cells was further induced, while Hsp27 and Hsp70 expression were also increased (Figure 6D). Then, cellular apoptosis in ASA + HS was inhibited significantly (*p* < 0.01), compared to HS (Figure 6E), and cytopathological examination showed that ASA treatment canceled the heat stress-induced cellular necrosis in spite of the retention of vacuolar and granular degeneration (Figure 6F). Meanwhile, oxidative stress in ASA + HS was also alleviated, marked by partial lower LPO plus MDA, and NO secretion was recovered (Figure 6G). These results suggest that ASA could induce Hsp90 to relieve the heat-stress damage of CMVECs.

### 3.7. Both Akt and PKM2 Signals were Strengthened by Hsp90 Induction Caused by ASA

**Figure 7:**
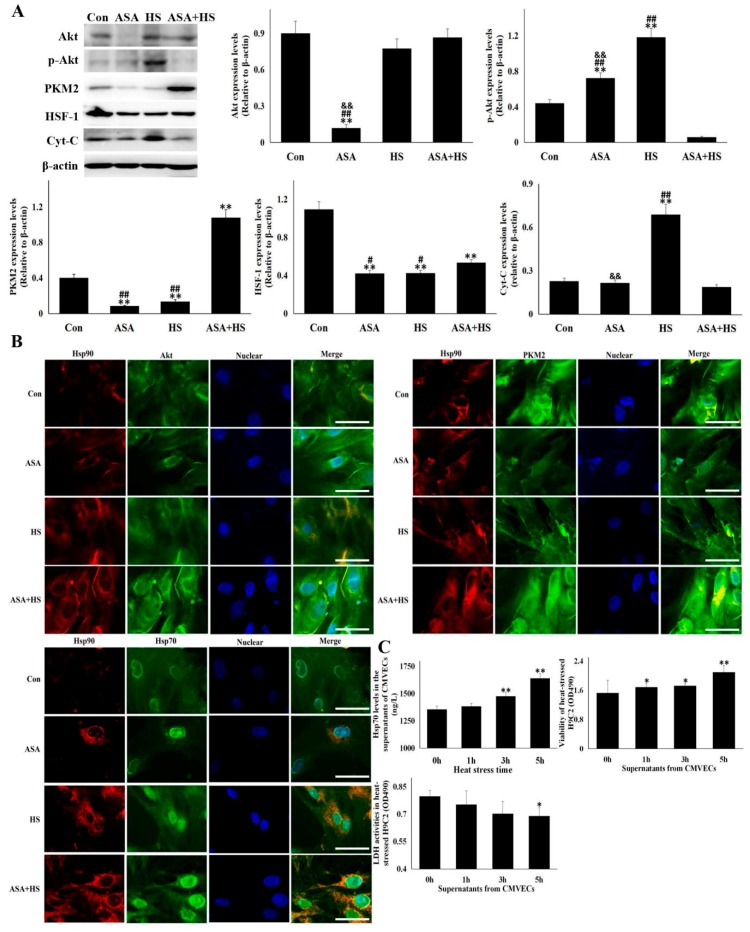
Akt and PKM2 signals and Hsp70 release from CMVECs were strengthened by Hsp90 induction. (**A**,**B**) CMVECs were treated with or without aspirin followed by heat stress for 5 h or not. Western blot analysis and immunocytochemistry were performed with the indicated antibodies. Data represent the means ± SD for three independent experiments. (**A**) Cellular total proteins were used for Western blot detection of the corresponding protein. The relative abundance of the tested proteins was normalized to that of β-actin. (**B**) Immunofluorescence was performed using anti-Hsp90α, anti-Akt, anti-PKM2, and anti-Hsp70 antibodies. Representative images from Con, ASA, HS, and ASA + HS are presented (Hsp90: red fluorescence, Akt/PKM2/Hsp70: green fluorescence, nucleus: blue fluorescence, the merged signals of Hsp90 with Akt/PKM2/Hsp70 in the cytoplasm: yellow fluorescence). Bar = 20 µm. Con, cells without aspirin treatment and heat stress; ASA, cells treated with aspirin; HS, heat-stressed cells without aspirin treatment; ASA + HS, heat-stressed cells following aspirin treatment. (**C**) CMVECs were heat stressed for different amounts of time, and then Hsp70 levels in the supernatant were detected with ELISA kit. The tested supernatants were added into cultured H9C2 cells, which were exposed to heat stress for 5 h. Cell viability and intracellular LDH levels were analyzed with CCK-8 and special kit, respectively. The differences of the data of cells with different treatment vs. that of the Con are indicated by * *p* < 0.05 and ** *p* < 0.01. The differences of the data of cells treated by ASA+HS vs. that in ASA or HS are indicated by **#** *p* < 0.05 and **##** *p* < 0.01. The difference of the data of cells treated by ASA vs. that in HS are indicated by & *p* < 0.05 and && *p* < 0.01.

The involvement of chaperone proteins of Hsp90 into resisting heat-stress injury was further validated (Figure 7). Aspirin caused a drop of Akt, but an explosion of p-Akt, in non-heat stressed cells, indicating that aspirin could effectively promote the functional phosphorylation of Akt to p-Akt. After heat stress, Akt levels in HS and ASA + HS were close, while p-Akt in ASA+HS was significantly lower than that in HS, implying that ASA also promoted the functional consumption of p-Akt during heat stress. For example, the heat stress-induced reduction of its downstream molecule, HSF-1, was reversed significantly (*p* < 0.05) in ASA + HS by aspirin treatment, compared to that in HS, thus further promoting the Hsp70 expression. As for PKM2, aspirin or heat stress alone decreased the expression of PKM2, but aspirin followed by HS treatment greatly exploded the PKM2 levels in heat-stressed CMVECs. ASA treatment also effectively inhibited the Cyt-C level in heat-stressed CMVECs, compared to cells exposed to heat stress alone. Immunofluorescence detection showed that the higher expression of Hsp90 by ASA significantly enhanced the co-localization signals of Hsp90 plus Akt, Hsp90 plus PKM2 and Hsp90 plus HSP70, and induced more aggregation of the merged signals of Hsp90 plus Akt and Hsp90 plus PKM2 around the cell membrane and nucleus. These data show that a higher Hsp90 expression from ASA could positively affect both Akt and PKM2 signals to protect CMVECs in heat stress.

### 3.8. Extracellular Hsp70 Release from CMVECs Inhibited Heat-Stressed Damage of Myocardial Cell Line, H9C2

A previous study showed that serum Hsp70 could protect myocardial cells from injury [28], however, its origin was still unclear. In this study, a preliminary attempt was made to analyze the extracellular Hsp70 source and its influence on cardiac parenchyma cells, myocardial cells. As shown in Figure 7C, Hsp70 was detected in the supernatant of non-treated CMVEC cultures and showed a significant and time-dependent increase under heat stress. Then, the supernatant of CMVECs exposed to heat stress or not was collected and used respectively to incubate the myocardial cell line, H9C2, followed by exposure to 5 h of heat stress. Data show that higher Hsp70 levels in the supernatant of CMVECs maintained a higher cell activity of the heat-stressed H9C2 cells, accompanied by a decrease of intracellular LDH. This implies that Hsp70 may be regulated by the Hsp90-Akt-HSF-1 axis and secreted out of CMVECs when heat stressed, and protects parenchyma cells (myocardial cells) in the heart from heat stress damage.

## 4. Discussion

The biological effects of heat stress on CMVEC cells were firstly investigated in this study, which showed that continuous heat stress could decrease the cell viability, injured cell architecture, and even induce cell apoptosis and necrosis. These adverse effects were intensified with the extension of heat stress time and probably involved oxidative damage attributed to heat stress due to increased cellular LPO and MDA levels. Under physiological conditions, the cellular redox status is balanced by enzyme and non-enzyme systems. However, certain stressful conditions such as heat stress increase the generation of ROS and/or decreases anti-oxidant capacity to enhance oxidative stress and synergistic augmentation of cell death [29,30]. Superfluous ROS cause oxidative stress injury to cellular compartments through lipid peroxidation and protein oxidation which has harmful implications on cellular physiological homeostasis, contributing to the capillaries’ relaxation dysfunction or obstruction [31,32]. Consistently, in this study, heat stress-induced endoplasmic reticulum and mitochondrial damage severely disturbed the NO synthesis and release of CMVECs.

Next, we observed that heat stress induced a significant increase of Hsp27, Hsp70 and Hsp90 levels, indicating that these Hsps play an important role in heat stress resistance, which is in agreement with our previous study [6,19]. However, their expressions were not enough for resisting heat-stress damage. We focused on the function of Hsp90 and then analyzed its client proteins. Increased Hsp90 effectively enhanced the interaction with Akt to maintain its abundance via inhibiting Akt degradation and increase its activation, thus providing tolerance against stress and controlling cell survival [33]. Chatterjee et al., reported that the PI3K-Akt signaling pathway stabilizes the expression of HSF-1, thereby controlling constitutive and inducible expression of Hsp70 in multiple myeloma cells [23]. We also found up-regulation of HSF-1 and Hsp70 upon substantial Akt activation during heat stress. Increased Cyt-C confirmed the damage of the mitochondrial structure and induced the apoptosis of CMVECs through the intrinsic mitochondrial pathway [34]. After inhibiting Hsp90 expression with SiRNA and then decreasing Akt activation, the levels of HSF-1 and Hsp70 were significantly down-regulated.

Unexpectedly, despite the Hsp90 induction and an improvement of co-localization with Hsp90, the PKM2 level was decreased under the heat stress-induced oxidative stress, which is in disagreement with a previous study on tumor cells [24]. Hsp90 knockdown reversed the down-regulation of PKM2. Therefore, it is plausible that PKM2 served as an urgent alternative pathway of PI3K-Akt when Hsp90 was insufficient for an adverse situation and that Hsp90 mainly regulated its activation and transposition rather than its intracellular level. Treatment of CMVECs with geldanamycin (Hsp90 function inhibitor) and/or triciribine (Akt activation inhibitor) resulted in the accumulation of PKM2 in cells to varying degrees, which verified our hypothesis above.

When Hsp90 was knocked down, CMVECs showed a more sensitive condition on heat stress, characterized by more serious injury. The potential interaction (co-localization) of Hsp90 with Akt and PKM2 was also reduced, thus directly weakening the protective action of the survival pathways, which was confirmed through pharmacological inhibition of Hsp90 and Akt in heat-stressed CMVECs. This is consistent with previous studies [23,24]. Interestingly, heat stress induced a slight restoration of HSF-1 level in SiRNA-treated CMVECs, which is responsible for the increase of Hsp27 and Hsp70 [23], implying that except for the Hsp90-Akt axis, HSF-1 might also be regulated by other mechanisms or influenced by other heat shock factors during heat stress [35,36]. This remains to be studied further.

Aspirin was verified to be a Hsp90 inducer in chicken myocardial cells through regulating HSF-1, HSF-2, and HSF-3 [21,22,37]. Considering that Hsp90 was indeed involved in the CMVEC cellular resistance of heat stress, aspirin was also used to test its effect on rat CMVECs. ASA was firstly proven to be safe for CMVECs. Some studies suggested that the acidity of organic acid such as ASA could stimulate nuclear translocation of HSF-1 and Hsps expression [38,39]. In this study, we explored the ability of ASA alone and in combination with equimolar sodium bicarbonate (ASA-Na) to induce heat shock proteins in CMVECs and found that ASA alone could justly stimulate the Hsp90 expression, while ASA-Na up-regulated all tested Hsps levels. This different result might be associated with the differences in cell or species types. After exposure to heat stress, CMVECs treated with ASA showed a better viability, lower oxidative levels, and normal NO release, compared to those in HS. In addition, the protective effect of ASA on heat-stressed CMVECs was closely connected with higher Hsp90 expression. When the cell was in hypoxia, Hsp90 could regulate intracellular homeostasis and was involved in maintaining vessel pressure through the NO mechanism [40,41,42].

In this study, Hsp90 over-expression induced by ASA significantly increased Akt activation in non-stressed CMVECs and the functional consumption of p-Akt, such as stimulating HSF-1 recovery in heat-stressed cells. However, the expression pattern of PKM2 was contrary to the p-Akt levels. These results further provided evidence that Akt activation is more sensitive to the fluctuation of the Hsp90 level, and that PKM2, as a standby pathway of PI3K-Akt, plays an urgent role in cell protection. The recovery of HSF-1 originated from ASA treatment in heat-stressed cells that regulated the high expression of Hsp27 and Hsp70, which in turn promoted the molecular chaperone function of Hsp90. Normally, inactive Akt stays mainly in the cytosol [43]. When the cell is stimulated, Akt is translocated to the plasma membrane where it is activated. Once activated, p-Akt translocates to the nucleus, where p-Akt positively regulates proliferation-associated factors and negatively regulates the expression of pro-apoptotic proteins by direct phosphorylation [44]. In addition, nuclear translocation of PKM2 activates β-catenin and also induces PKM2 expression by splicing the PKM pre-mRNA into PKM2 mRNA [45,46]. Nuclear PKM2 acts as a transcriptional co-activator of hypoxia-inducible factor 1-alpha (HIF-1α) to reprogram cell metabolism [47]. It is worth noting that ASA also drove the merge signals of Hsp90 with Akt and PKM2 to the cell membrane and perinuclear area, which were essential for the activation of client proteins to maintain membrane completeness and expression of cytoprotective genes in the nucleus.

An inflammatory response after open heart surgery can stimulate Hsp70 expression and a release of vascular endothelial cells to the circulatory system through cell secretion and cell destruction [25,48]. It has been proven that peripheral Hsp70 can activate the expression and release of downstream NF-κB-mediated inflammatory mediators through the Toll-like receptors, the classical ligand of lipopolysaccharide in myocardial cells [49]. The present study found evidence that heat stress could also cause the release of highly expressed Hsp70 in CMVECs to the cell supernatant, and external Hsp70 exerted a protective effect on heat-stressed myocardial cells. Although the action mechanism of Hsp70 in the supernatant on myocardial cells needs to be further studied, its release seems to be closely correlated with the positive regulation of the Hsp90-Akt-HSF-1 axis and heat-stress damaged cytomembrane.

## 5. Conclusions

**Figure 8:**
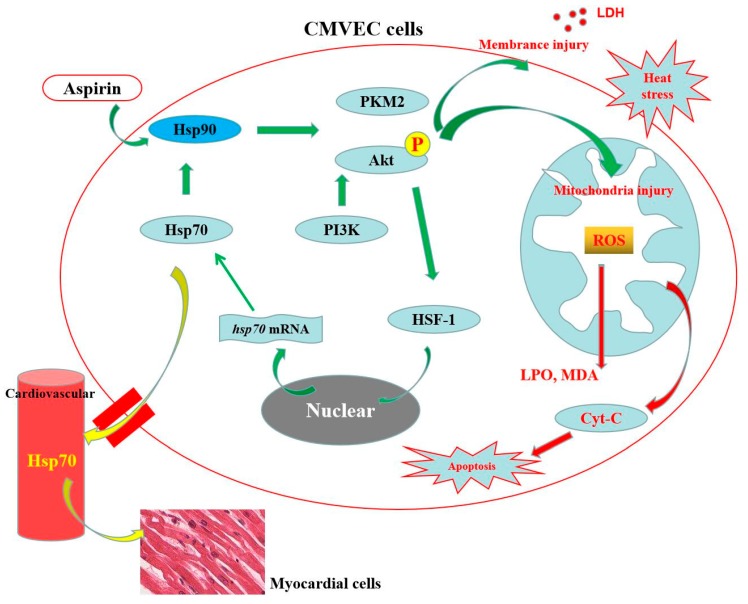
Schematic representation of the Hsp90-mediated resistance to heat stress damage in the heart. Heat stress caused the oxidative stress and cytomembrane damage, thus resulting in apoptosis and necrosis of CMVECs. Hsp90 mediated Akt and PKM2 signals differently through their interaction to inhibit heat stress damage. In addition, Hsp90-mediated Akt activation regulated Hsp70 expression and release via HSF-1 to protect myocardial cells. Aspirin could improve the resistance of CMVECs to heat stress through inducing Hsp90 expression and its promotion of Akt and PKM2 function.

As illustrated in Figure 8, our data indicate that CMVEC cells were severely injured after heat stress, mainly in the cytomembrane and mitochondrion, and induced oxidative stress and cellular dysfunction. Cellular resistance to heat stress was closely associated with the signals of Hsp90-Akt and Hsp90-PKM2, which functioned under a complementary situation. Akt promoted HSF-1 to regulate Hsp70 expression, thus in turn strengthening Hsp90 function. Our study also provides, for the first time, evidence of aspirin being involved in Hsp90 induction-mediated protective pathways in heat stress-damaged CMVE cells, and a viewpoint of extracellular Hsp70 regulated by the Hsp90-Akt-HSF-1 axis of CMVECs to protect heat-stressed myocardial cells.
